# Perception of the Saudi Population on Abortion Decisions in Congenital Fetal Anomalies

**DOI:** 10.7759/cureus.32587

**Published:** 2022-12-16

**Authors:** Rahaf H Alharbi, Lujin Alajmani, Raghad K Alhajrasi, Mawdah O Hindi, Basim S Alsaywid, Miltiades D Lytras

**Affiliations:** 1 College of Medicine, King Saud Bin Abdulaziz University for Health Sciences, Jeddah, SAU; 2 Pediatric Urology Section, Department of Urology, King Faisal Specialist Hospital and Research Centre, Riyadh, SAU; 3 Education and Research Skills Directory, Saudi National Institute of Health, Riyadh, SAU; 4 Computer Science, Effat College of Engineering, Effat University, Jeddah, SAU

**Keywords:** abortion in ksa, women, religion, religion and culture, induced abortion, abortion, congenital anomalies

## Abstract

Introduction

Termination of pregnancy for fetal anomalies is well reported in the literature and accepted by the western and other non-Muslim communities, but Muslim communities’ perception is poorly reported and rarely mentioned. This study aims to evaluate the perception of the Saudi community on abortion decisions as a management option in congenital fetal anomalies.

Methods

This is an observational, descriptive cross-sectional study, where participants of Saudi nationality, living in Jeddah, and consenting to participate in the research filled up a self-administrated, structured, close-ended, validated questionnaire. The level of agreement was measured on a Likert scale.

Results

A total of 574 participants were included in the study; 43.3% were female. The mean age of the participants was 30.3 years (SD = 10.6). Undergraduate students were 58.9%, single participants were 56.3%, and participants without children were 61.3%.

The prevalence of abortion was 17.9%. The overall agreement on accepting abortion as an option was 61%. Gender (p<0.001), knowledge level (p=0.003), and religion (p=0.01) were the most important factors that influenced people’s perception of abortion. Other factors like participants’ age (p=0.09), level of education (p=0.48), marital status (p=0.16), having children (p=0.48), and gender of the fetus (p=0.2) were not significant factors in their decision to choose abortion.

Conclusion

Overall, Saudis were more inclined to accept abortion in case of a confirmed congenital anomaly, yet females were more accepting of the idea than males.

## Introduction

Abortion is the act of terminating a pregnancy; it could be either induced or spontaneous. Spontaneous abortion is most referred to as miscarriage, and it occurs with no intention of terminating the pregnancy. On the other hand, the deliberate termination of pregnancy, known as induced abortion, is performed either by surgery or by the usage of certain medications [1].

People’s perception regarding abortion differs greatly throughout the world. The basis of this variation in decision-making is due to socioeconomic, medical, and religious differences [2]. There are several reasons behind the decision to intentionally end a pregnancy, such as potential risk to a woman’s life or physical and mental health, rape, and fetal impairment [2]. Abortion in Saudi Arabia is a very complex process to go through, especially if the abortion was due to fetal anomalies. Moreover, induced abortion needs to be approved by many authorities such as consultants in the ethical committee of the hospital. Furthermore, the criteria for approval can differ from one hospital to another. In addition, there are no specific clear roles or bylaws regarding abortion in Saudi Arabia. The general rule is “Abortion is prohibited except in critical conditions” and each hospital defines and decides its own criteria for “critical conditions.”

Termination of pregnancy for fetal anomalies is well-reported in the literature and accepted by the westernized community, but Muslim communities’ perception is poorly reported and rarely mentioned [3,4]. This is mainly because the vast majority of Muslim governments rule by the laws of Shari'a that highly prohibit abortion, except in critical cases that threaten the mother’s life. However, some Muslim countries like Turkey and Tunisia have legalized induced abortion [2]. 

The prevalence of fetal anomalies in Saudi Arabia is not very well researched; however, due to increased rates of consanguinity and maternal age, the percentage is expected to be high. In a local study, the prevalence of major congenital anomalies was 46.5 per 1000 live births [5].

The recent developments in methods used for the early detection of fetal anomalies, such as ultrasonography, have increased within the last decade [6]. Because of the capability of diagnosing congenital anomalies early, the number of abortions has risen compared to the past. About 2% of total abortions according to recent literature are related to congenital fetal anomalies [7].

The given cultural context in Saudi Arabia and the current regulations and procedures related to abortion challenge the study of the phenomenon. Our study provides a significant contribution to understanding key facts for abortion in congenital fetal anomalies in Saudi Arabia. It also investigates complementary aspects of the cultural determinants of the phenomenon offering knowledge to the international research community.

Our study investigates social aspects of the abortion phenomenon in Saudi Arabia, aiming to interpret significant cultural dimensions of the phenomenon as well as document the perceptions of individuals towards this sensitive case. The key research question for our research approach is “Do Saudis accept induced abortion as a management option in fetuses with congenital anomalies?” Our null hypothesis is that Saudis do not accept induced abortion as a management option in fetuses with congenital anomalies.

The study objectives are to evaluate the perception of the Saudi population regarding abortion decisions in cases of congenital anomalies and to determine the factors that have a significant impact on the mother’s decision of rejecting induced abortion despite the presence of fetal anomalies that will result in a poor quality of life for her child.

## Materials and methods

Research study design 

This is an observational, descriptive, cross-sectional study design, where all participants who fulfilled the inclusion and exclusion criteria have been invited to fill up a self-administered, close-ended, structured, and internally reviewed questionnaire that was available in two languages, Arabic and English, to explore the perception of Saudi society on the usage of induced abortion for congenital fetal anomalies in Jeddah, from 2019 until 2021.

Identification of study participants

The target population was those who fulfilled the inclusion criteria, that is, adults >18 years old, of both genders, Saudi citizens, and those who lived in Jeddah city during the survey period and were willing to participate in filling up the questionnaire after approving the consent form. Through a non-probability, convenience sampling technique, the study population was selected from the assigned criteria of our target population with a preplanned calculated sample size of 389 participants to be included in the final analysis according to the calculation done by a biostatistician at our university based on the population of the city of Jeddah. Initially, Jeddah was divided into three major sections, Northern, Southern, and Eastern. One to two public places have been selected representing each section, with overall six public places included. We approached our target population who were visiting major shopping malls like Red Sea Mall, Alyasmeen Mall, Alsalam Mall, and other public places like the Jeddah waterfront in those pre-selected areas.

Data collection process

All subjects who agreed to participate in our study filled up a self-administered, close-ended, structured, and validated questionnaire. The questionnaire was created by the research team, who validated the construct, and face validity was conducted by experts from KAIMRC (King Abdullah International Medical Research Center) to ensure that it effectively captured the investigated topic and did not contain any errors. The questionnaire is composed of three sections. The first section is about the basic demographic features of our study population. The second section measured the participant’s knowledge regarding induced abortion and its related topics. The third section targeted the perception of our participants in selecting abortion as a management approach. The questionnaire was piloted and showed high reliability with Cronbach's alpha calculated at over 0.8.

 The participants answered questions on a Likert scale where they chose the degree to which they agreed to specific statements by choosing from the following answers (Strongly Agree-Agree-Neutral-Disagree-Strongly Disagree).

Data analysis

SPSS software (IBM Corp. Released 2020. IBM SPSS Statistics for Macintosh, Version 27.0. Armonk, NY: IBM Corp) was used for data entry, data management, and data analysis. A simple descriptive analysis was performed for qualitative data, frequency and percentage were reported, and for quantitative analysis, mean and standard deviation or median and interquartile range were reported for normally distributed data or skewed data, respectively.

For bivariate analysis, to compare the gender of the participant and the perception of abortion, a chi-square test was used, with the two-tailed and significant level set at 0.05. An independent sample student t-test was reported for other contributing factors on the level of knowledge, the strength of religious beliefs, and age. Logistic regression analysis was performed to evaluate the significance of multiple factors on the agreement of abortion decisions.

A scoring system was done to measure the knowledge of fetal anomalies and abortion and to correlate it with their opinions regarding different aspects of abortion. The scoring system was based on several close-ended questions that explored people’s comprehension of the difference between induced and spontaneous abortion, the experience of abortion, people’s awareness of current laws of abortion, the types of tests done to detect fetal anomalies in Saudi Arabia, and people’s expectations of how common congenital anomalies are in Saudi Arabia.

This study was approved by the Institutional Review Board (IRB) of King Saud Bin Abdulaziz University for Health Sciences, with Approval Study Number SP19/122/J, dated April 24, 2019.

## Results

Demographics

In our study, a total number of 574 respondents participated, meeting the inclusion criteria presented in the previous section. In total, 249 of them (43.3%) were females and 325 (56.6%) were males. The mean age of the participants was 30.3 years (SD = 10.6) with ages ranging between 18 and 80 years old. The mean age of male participants was 30.5 (SD = 12.15), and the mean age of females was 30.1 years (SD = 9.26); the difference was not statistically significant (p = 0.66).

Most participants were undergraduates (58.9%; n= 338) followed by secondary/high school graduates (30.1%; n= 173); postgraduate participants represented 7.7% (n=44), intermediate school graduates represented 2.4% (n= 14). and elementary school graduates represented 0.9% (n=5).

Our sample population consisted largely of single participants (n= 323) representing (56.3%) married participants (n=228) representing (39.7%), divorced individuals (n=19) representing (3.3%), and finally, widowed participants (n=4) representing only (0.7%) of the total sample size. Most of our study participants did not have children (n=352) representing (61.3%) while the others who had children represented (38.7%) with a total number of 222. The mean score for their religious beliefs was 7 out of 10 as a maximum score (SD = 2.1).

The prevalence of abortions in our study population was 17.9% (n=103), both spontaneous and induced.

According to the research results, 312 (54.4%) participants agreed on accepting abortion in case of fetal anomalies, 95 (16.6%) disagreed, and 167 (29.1%) were neutral. In the female population, 61.8% (n=201) agreed on abortion in case of confirmed congenital anomalies and 12.9% (n=42) disagreed. However, males’ perception was significantly different as only 44% (n=111) of males agreed while males who disagreed represented 21% (n=53). This difference in opinion between genders was statistically significant (chi-square = 17.5, df=2, p<0.0001). Participants’ age was not a significant determinant of their agreement on abortion; the mean age for agreement was 29.4 years, 31.3 years for neutral, and 31.4 years for disagreement (analysis of variance (ANOVA), F = 2.34, df=2, p = 0.09). Similarly, the agreement on abortion was not altered by the level of education of our participants (chi-square = 7.5, df=8, p = 0.49). Fifty percent of married participants agreed on abortion in comparison to 57% of single and 63% of divorced; this difference was not statistically significant (chi-square test = 9.3, df=6, p = 0.15). Additionally, participants with a high score on religious beliefs disagreed with abortion more than participants with a lower score (F=4.51, df=2, p = 0.01).

Most of the participants (90.1%; n=517) believed that the gender of the fetus does not play a role in the decision of abortion. A total number of 312 participants (54.4%) agreed that the mother should be allowed to undergo induced abortion while 95 participants (16.6%) did not support the mother’s freedom to undergo an abortion. Nonetheless, 167 (29.1%) were indifferent.

In addition, study participants were asked to choose the most fundamental factors that have a significant impact on the mother’s decision to undergo an abortion. The results varied, however, the most prominent factors were religious and psychological factors (Table 1).

**Table 1:** Factors affecting women's decision on abortion

Effectors on mother’s abortion decision	N	Percent
Religious causes	345	25%
Family pressure	127	9.3%
Complicated official protocols	109	7.9%
Lack of abortion specialized and authorized centers	104	7.6%
Cultural causes	101	7.4%
Personal reputation	78	5.7%
Psychological	343	25%
Social pressure	165	12%

Participants who did not have children are leaning toward accepting the idea of induced abortion more than those with children. However, the possession of children does not impose a statistically significant effect on people’s opinions regarding the termination of the pregnancy with a p-value of 0.47 (Table 2).

**Table 2:** Correlation between having children and people's acceptance of abortion

			Agree	Neutral	Disagree
Do you have children?	Yes	Count	110	71	41
		% Within do you have children?	49.5%	32%	18.5%
	No	Count	118	57	33
		% Within do you have children?	56.7%	27.4%	15.9%
	Not married	Count	84	39	21
		% Within do you have children?	58.3%	27.1%	14.6%
Total		Count	312	167	95
		% Within do you have children?	54.4%	29.1%	16.6%

A great proportion of both genders agreed that mothers who have undergone abortions need emotional support, with females representing 80.3% and males 72%. A minority of the population, only 3.4% of females and 5.6% of males, thought that there is no need for emotional support.

A scoring system was created to measure people’s level of knowledge of fetal anomalies and abortion, and to correlate it with their opinions regarding different aspects of abortion. Based on the results, a better understanding of congenital anomalies and abortion, including screening tests, procedures, and laws greatly impacted peoples’ acceptance of induced abortion of fetuses with congenital anomalies (F=5.9, df=2, p = 0.003).

To evaluate the significant interaction of multiple factors, (gender, level of knowledge, and strength of religious beliefs) on the agreement of abortion decision, a logistic regression procedure (LRP) was performed. Prior to interpreting the results of LRP, several assumptions were evaluated. Assumption testing conducted prior to the analysis did not indicate any violation. The omnibus model for the logistic regression analysis was statistically significant, chi-square (df=3, N=574) = 27.18, p<0.0001, Cox & Snell R2 = 0.046. The Hosmer and Lemeshow test results confirmed that the model was a reasonable fit for the data (chi-square (df=8, n=574) = 12.17, p = 0.144).

Gender (b=0.62, SE=0.17, p<0.0001) and level of knowledge (b=-0.013, SE=0.005, p = 0.007) were the most significant factors that improved the model’s predictive capability. The level of religious beliefs did not appear to significantly influence the probability of participants’ agreement on the decision of abortion.

## Discussion

Abortion (termination of pregnancy for fetal anomalies) is well-reported in the literature and accepted by the westernized community. On the other side, abortion is a controversial and highly sensitive subject, especially in religious communities, such as Saudi society. An extremely scarce number of studies have tackled this topic. In the broad international literature on the phenomenon, various complementary aspects provide significant insights for the justification of the research problem of our study (Table 3). The value-based approach to abortion in the literature also covers aspects related to Indicators for therapeutic abortion and understanding the implications of induced abortion. The investigation of attitudes related to abortion was discussed in a recent study in the US and South Africa [8,9]. The main deployed method included the relation of abortion acceptability (in cases of poverty and fetal anomaly) with attitudes about social welfare programs and gender roles. The relevant research also assessed differences by race/ethnicity and education characteristics. In our study, this is a significant determinant of our study problem. We are interested in understanding the attitudes among the Saudi population toward abortion in congenital fetal anomalies. This aspect of the phenomenon is significant since there is a lack of similar studies in KSA.

**Table 3:** An overview of our critical literature review and key aspects of the research problem

		Literature review		
Reference	Year	Analysis/Method	Methods/Focus	Impact on our research
[9]	2020	Data from the U.S. General Social Survey and South African Social Attitudes Survey.	Focus: to measure whether abortion acceptability (in cases of poverty and fetal anomaly) is related to attitudes about social welfare programs and gender roles, then assessed differences by race/ethnicity and education.	Connection of abortion to attitude/ race/ethnicity/education differences
[10]	2020	Survey/Literature Analysis	Focus: to determine the factors that help or hinder accessibility and sustainability of abortion services in England.	Factors enabling abortion accessibility
[11]	2018	130 women with a history of abortion after a diagnosis of a fetal anomaly in German.	Focus: in psychometric properties of a measure of stigma among women who had had an abortion after a diagnosis of fetal anomaly. Analysis: psychometric properties of the ILAS scale and Individual and situational factors associated with stigma.	Psychometric studies
[12]	2018	127 health facilities with PAC capacity, 227 women seeking abortion-related care, and 118 experts knowledgeable about abortion in Zimbabwe.	Focus: On the national provision of PAC, the first-ever national incidence of induced abortion, and the proportion of pregnancies that are unintended. Analysis: The Abortion Incidence Complications Method (AICM) indirectly estimates the incidence of induced abortion by obtaining a national estimate of PAC cases.	Connection of abortion and unintended pregnancies
[13]	2018	196 eligible Medical Termination of Pregnancy (MTP) seekers at a tertiary care teaching hospital in North India.	Focus: on the psychological assessment of legal abortion. Analysis: Risk factors included primipara, second-trimester abortion, MTP on humanitarian grounds (rape), fetal congenital anomalies, and maternal illness.	Psychological assessment of legal issues in abortion risk factors
[14]	2018	147 women whose TOPFA dated back 1 to 7 years.	Focus: On the influence of perceived and internalized stigma on women’s long-term adjustment to a TOPFA. Analysis: The associations between perceived stigma at the time of the TOPFA, current internalized stigma, and symptoms of grief, trauma, and depression.	Relation to perceived stigma.
[15]	2018	14 consultants in fetal medicine, obstetrics, neonatology, and pediatrics) and social care professionals (nine individuals with roles supporting people living with impairment) from the Northeast of England.	Focus: on the perspectives of professionals around the issue of termination of pregnancy for non-lethal fetal anomaly (TOPFA). Analysis: an inductive thematic approach facilitated by NVivo.	Perspectives of professionals
[16]	2018	Patient-centric analysis	Focus: On the indications for therapeutic abortion and to discuss the legal implications of this procedure in Romania. Analysis: blood tests, fetal morphology scans, amniocentesis as well as a complete survey of the patients’ medical history.	Indicators for therapeutic abortion
[17]	2018	78 chorionic villus specimens from early spontaneous abortion patients with no obvious abnormality are collected after microassay analysis. 60 chorionic villus specimens from induced abortion patients with no obvious abnormality	Focus: On submicroscopic chromosomal imbalances and how they contribute to early abortion. Analysis: The submicroscopic structures of chromosomes from two groups using an array-based comparative genomic hybridization (aCGH).	Understanding the factors causing an early abortion
[18]	2020	351 pregnancies in Switzerland.	Focus: On high abortion rates and if these might relate to specific characteristics of women with DS, their life situation, and the course of pregnancy. Analysis: personal characteristics and pregnancy complications that might increase the probability of abortion of women with DS, women with other forms of intellectual disability (ID), and women without ID.	Connections of personal characteristics and abortion rates
[19]	2019	39 French PD centers.	Focus: on the practice of feticide in second- and third-trimester termination of pregnancy for fetal anomalies (TOPFA).	Understanding causal factors of the phenomenon
[20]	2019	Meta-Analysis of existing data	Focus: on determining whether a physician’s procedure volume influences complications after induced abortion. Analysis: population-based retrospective data on surgically induced abortion procedures in Ontario between 2003 and 2015.	Understanding the implications of induced abortion
[21]	2020	Position paper	Focus: on high-quality abortion services and how they should offer women a choice between abortion methods and provide the one they prefer.	Analysis of the women's preferences for modern abortion methods
[22]	2018	325 abortion experts like researchers, providers, funders, policymakers and advisors, advocates. 424 peer-reviewed literature.	Focus: on a new conceptual framework for studying trajectories to obtaining abortion-related care. Analysis: all the known factors influencing a trajectory.	Trajectories of womens’ abortions

Additional insights related to factors enabling abortion accessibility [10] and sustainability challenge the research design of our study, bringing forward the need to investigate the requirements for a sustainable abortion in the congenital fetal anomalies management approach. This is a key step toward the support of mothers and enhancing the impact on society and sustainable development in KSA.

The relevant literature on abortion considers also psychometric studies for abortion issues and psychological assessment. These studies, together with complementary ones on the relationship of abortion to perceived stigma, constitute a sophisticated psychological background and context for the study of the phenomenon that we want to approach and exploit in our research design. Additional studies related to the study of risk factors associated with abortion and the perspectives of professionals provide indicative aspects for the social aspects and the implications of abortion.

Women’s ability to make a decision on abortion is influenced by many factors, such as education level, emotions, and social and religious pressure (modern populations are more open to such a decision unlike traditional and conservative communities) [4]. The mere notion of abortion is controversial in Saudi society due to its sensitivity; therefore, most individuals tend to avoid this subject. To the best of our knowledge, there are no studies addressing the perception and acceptability of performing an abortion on fetuses with congenital anomalies in our community. Our study aimed to investigate the perception of the Saudi population regarding abortion in cases of congenital anomalies.

We were interested to evaluate the determinants that impacted mothers' decision to reject induced abortion despite the presence of fetal anomalies that will result in a poor quality of life for their child, and it was found that the greatest proportion of study participants believed religious and psychological factors to have the greatest impact, followed by social and family pressure, complicated official protocols, lack of specialized and authorized abortion centers, cultural causes, and worry about personal reputation. Meanwhile, a study done in 2018 in Mozambique revealed that the most important factors that influence abortion decision-making among young women mainly ranged between 1) lack of women’s autonomy to make their own decisions regarding undergoing an induced abortion, 2) their general lack of knowledge, 3) the scarcity of local abortion services, and 4) the powerful influence of healthcare providers on decision- making [23].

Interestingly, the results of our study/our study revealed that females are more inclined to accept the idea of terminating a pregnancy in case of confirmed fetal anomalies. Similarly, a study done in Pakistan also found that women had a more favorable attitude toward induced abortion [24]. The impact that the level of education has on people’s opinions about the most prominent factors that could potentially affect the mother’s decision of undergoing abortion varied significantly. Participants with higher educational levels (high school graduates and beyond) tended to choose religious and psychological aspects as the main contributing factors to this decision. Meanwhile, most participants from all educational levels thought that the rest of the factors, including social, cultural, and family pressure, personal reputation, lack of specialized abortion centers, and complicated apportion protocol, played no fundamental role in a mother’s abortion decision [25].

Policy-making recommendations

It is important to promote some policy-making recommendations and guidelines as a reflective mechanism for the integration of research-based evidence to practice.

In Table 4, we summarize the key findings of our study, and we also provide initial policy recommendations and aspects of an action plan in KSA.

**Table 4:** Experience of abortion by the participant or his partner

Key Findings	Policy Recommendation	Value-Based Approach
Prevalence of abortion in KSA	The prevalence rate requires a systematic review of policies and procedures related to abortion in congenital fetal anomalies	Redesign of procedures, value realization analysis
The fundamental factors with an impact on the mother’s decision to undergo abortion	Seminars and awareness campaigns for abortion procedure refinement, e-Psychological support, and the development of specialized centers	Trusted knowledge, awareness campaigns, e-counseling infrastructures
Public health implications	Abortion data observatory official monitoring and reporting, transparent procedures, information campaign	Data observatory, dashboard reporting, policies refinement, workflow, and support
Vision 2030	Digital transformation in abortion procedures, digital hub for information resources, counseling for abortion, e-service, vibrant and prosperous society	Digital transformation, digital hub

Prevalence of abortion

Our study, with its given limitations, provides useful data and input for understanding the prevalence of abortion. Without focusing on the absolute number of abortion rates as measured in our study, we need to communicate that abortion is an existing phenomenon with various sociocultural, psychological, and other determinants. As such, it requires a systematic review of policies and procedures related to abortion due to congenital fetal anomalies. This will effectively support the design of new procedures that will allow a value-based approach to abortion due to congenital fetal anomalies. In the same context, fundamental factors with an impact on the mother’s decision to take a decision need to be analyzed further and to be the basis for support activities.

Fundamental factors with an impact on a mother’s decision to undergo abortion

Our study revealed significant hermeneutic factors for abortion while several of them have also been found in the relevant literature. An initial study of these factors leads to significant propositions for supporting mothers. The first is related to the need for a new educational and awareness campaign for abortion in congenital fetal anomalies. It is necessary to utilize international scientific knowledge with respect to the cultural context in KSA to offer trusted, accurate, and reliable training interventions, including micro-courses, micro-content campaigns in social media, and awareness events in KSA.

Another significant finding is related to the official procedures related to abortion. According to the participants in our survey, these procedures were highlighted as a key determinant of the phenomenon with emphasis on the need for simplification and digital transformation. Our recommendation is a significant refinement and revision of procedures related to abortion in congenital fetal anomalies as a critical step for the support of mothers. We also recommend the utilization of Vision 2030 and its mandate related to Health and Digital Transformation in KSA in the direction of establishing e-channels and electronic workflows for the utilization of the process.

Our survey also revealed the significance of the psychological factors and their impact on the mother’s mental health condition. We recommend the establishment of e-psychological support and e-counseling services for mothers with reference to the international standards for relevant services.

Finally, the development of specialized centers with expertise on abortion in congenital fetal anomalies will also provide significant value to a new value-based approach to abortion in KSA.

Public health implication

Our study also contributes to the monitoring of public health policies and procedures. It is an initial input for understanding the impact of abortion on congenital fetal anomalies in KSA. We recommend the establishment of a transparent trusted observatory for abortion figures and facts in KSA as an official monitoring and reporting mechanism aiming to provide value-based insights into the prevalence of the phenomenon in KSA toward enhanced decision-making capability. Furthermore, the redesign of transparent procedures and an integrated strategy for a robust knowledge and awareness campaign are additional initial recommendations from the evidence of our study.

Vision 2030 and digital transformation in health

In another direction, our study is also connected and provides interesting insights into a greater context, such as Vision 2030, and the relevant strategic plan for digital transformation in health. We recommend the digital transformation in abortion procedures as a bold response to the existing prevalence of the phenomenon in KSA. This will secure significant resources and will build trust, transparency, reliability, and responsibility for the various stakeholders. We also recommend the design and implementation of a digital hub for information resources and counseling digital services as a key requirement for a vibrant society and prosperous nation.

Toward this direction, we recommend the development of a senior scientific task force, consisting of physicians, policymakers, and government officers that:

1. Standardize Saudi maternal-fetal specialist guidelines on identifying congenital fetal anomalies and the type of fetal anomalies amenable for abortion currently widely accepted at the ethics committees of hospitals.

2. Standardize the policies and protocols for the promotion of the health of mothers undergoing the phenomenon.

3. Recommend infrastructure developments adopting the latest technological evolution.

4. Propose new systems for psychological support and awareness related to the phenomenon.

5. Set up guidelines for digital hubs and platform-enabled environments that will facilitate the procedure.

Our research is the first attempt and one of the first studies in KSA for the study of abortion in congenital fetal anomalies.

Limitations

This study was limited by the small sample size of participants who agreed to fill up the questionnaire, which increases the chance of selection bias. Participants were reluctant to fill up the questionnaire due to the sensitivity of the topic. Moreover, the study was conducted in one city, and it was not a nationwide survey. During the data collection period, the COVID-19 pandemic restricted the research team to conduct the community survey properly. On the other side, the limitations of the study provide fertile ground for further research as indicated in the previous section. We consider this study as a pilot study with significant findings and value that will be expanded and utilized on a nationwide survey that we will deliver in Fall 2022 aiming to publish the results in 2023.

## Conclusions

Our study approached a significant phenomenon related to public health, with sensitive determinants given the sociocultural context in KSA. The study of abortion in congenital fetal anomalies provided significant insights related to the objectives of this study. Saudis were more inclined to accept abortion in case of a confirmed congenital anomaly, but females were more accepting of the idea. However, couples who had children were leaning more toward rejecting the option. The most significant factors that played a role in the decision-making on abortion include gender, religious aspects, educational level, and being a parent of children.
